# Cluster of differentiation 147 is a key molecule during hepatocellular carcinoma cell-hepatic stellate cell cross-talk in the rat liver

**DOI:** 10.3892/mmr.2015.3429

**Published:** 2015-03-04

**Authors:** TIANYOU MA, ZHILUN WANG, ZHANTIAN YANG, JINGHONG CHEN

**Affiliations:** Institute of Endemic Diseases, Environment Related Gene Key Laboratory of Ministry of Education, Xi’an Jiaotong University, Xi’an, Shaanxi 710061, P.R. China

**Keywords:** cluster of differentiation 147 molecule, hepatocyte, hepatic stellate cell, cross-talk

## Abstract

The cross-talk between hepatocellular carcinoma (HCC) cells and activated hepatic stellate cells (HSCs) is considered to be important for modulating the biological behavior of tumor cells. However, the molecular links between inflammation and cancer in the activation of HSCs remain to be elucidated. The present study demonstrated that cluster of differentiation (CD)147 is a key molecule involved in the interaction between HCC cells and HSCs. The effects of conditioned medium from human HCC cells on the activation of the human HSC line, LX-2, were assessed using a 3-(4,5-dimethylthiazol-2-yl)-2,5-diphenyltetrazolium bromide assay, western blotting and reverse transcription-quantitative polymerase chain reaction (RT-qPCR). Western blotting, RT-qPCR and gelatin zymography were also used to investigate the effects of CD147 on the activation of LX-2. The expression levels of α-smooth muscle actin (α-SMA) and CD147 were assessed in a co-culture system of LX-2 and FHCC-98 cells by immunofluorescence staining and immunoblotting. In hepatic tissues from a rat model of fibrosis, immunohistochemistry and immunoblotting were performed to detect the expression levels of α-SMA and CD147 Tumor-conditioned medium and CD147 promoted cell proliferation, activated LX-2 cells, increased the expression levels of α-SMA, collagen I and tissue inhibitor of metalloproteinase-1 (TIMP-1), and increased the secretion of matrix metalloproteinase (MMP)-2. The HSCs, which were induced in the co-culture system of HCC cells and HSCs exhibited marked expression levels of CD147. In the hepatic tissue of rat models of fibrosis induced by CCl_4_, marked expression levels of CD147 were observed in the activated HSCs. Therefore, CD147 promoted the activation of HSCs and was a key molecule during HCC cell-HSC cross-talk in the rat liver.

## Introduction

Hepatocellular carcinoma (HCC) cells commonly develop following liver cirrhosis and fibrosis (1). Liver fibrosis is considered a premalignant disease, which occurs initially during pathological changes of the liver (2,3). During chronic liver damage, hepatic stellate cells (HSCs), the predominant type of hepatic non-parenchymal cells, transdifferentiate into extracellular matrix-producing myofibroblasts and are activated (4). The activated HSCs proliferate and migrate towards the area of tissue remodelling, secreting extracellular matrix (ECM) proteins and growth factors, and providing an important microenvironment for hepatic regeneration (5,6), which are vital for hepatocellular function and the response of the liver to injury (7). Therefore, activated HSCs are the predominant source of ECM proteins in liver fibrosis, on which HCC commonly develops, and serves as an important mediator in the inflammation-fibrosis-carcinoma axis and in tumor metastasis (7,8).

There are apparent interactions between HCC cells and HSCs during normal physiological processes and pathological changes of the liver. The majority of investigations have focused on the importance of HSCs in the development of HCC and revealed that HSCs stimulate the invasion and migration of HCC cells, promoting the tumorigenicity of HCC cells (9–11). Previous studies have demonstrated that HCC cells secrete cytokines, which promote the activation of HSCs (12–14). The cross-talk between HCC cells and the surrounding microenvironment is considered to be important in modulating the biological behavior of the tumor. However, the molecular mechanisms, which connect inflammation and cancer in the activation of HSCs remain to be fully elucidated.

The present study investigated the cluster of differentiation (CD)147 molecule, which is markedly expressed in HCC cells. It is a key factor in the activation of HSCs and is an important molecule during HCC cell-HSC cross-talk. The aim of the present study was to investigate the mechanism underlying the interaction between HCC cells and the surrounding microenvironment, with a particular focus on the role of HCC cells in modulating the biological activities of the HSCs.

## Materials and methods

### Reagents

Dulbecco’s modified Eagle’s medium (DMEM) was purchased from Hycylone (Logan, UT, USA) and fetal bovine serum (FBS) was purchased from Sijiqing Biological Engineering Materials (Hangzhou, China). TRIzol reagent and goat anti-mouse secondary-antibodies conjugated with Alexa Fluor 594 (cat. no. A-11005) or fluorescein isothiocyanate (FITC; cat. no. A16079) were purchased from Invitrogen Life Technologies (Carlsbad, CA, USA). ReverTra Ace-a-TM was purchased from Toyobo Co., Ltd, (Osaka, Japan) and a mouse monoclonal antibody against α-smooth muscle actin (α-SMA) (cat. no. ab7817) and CD147 (cat. no. ab78106) were purchased from Abcam (Cambridge, UK). Goat anti-mouse secondary antibodies, conjugated with horseradish peroxidase (cat. no. BA1004), were purchased from Boster, Ltd. (Wuhan, China).

### Cell culture and collection of HCC-conditioned medium (CM)

The FHCC-98 human HCC cell line and the LX-2 human HSC line (5×10^5^ cells/100 mm culture dish) (Department of Cell Biology, Fourth Military Medical University, Xi’an, China), were cultured at 37°C in a humidified atmosphere, containing 5% CO_2_ in DMEM, containing 5% FBS, 100 U/ml penicillin and 100 mg/ml streptomycin (HyClone Laboratories, Inc., Logan, UT, USA).

For co-culture, the FHCC-98 and LX-2 cells (5×10^5^ cells/6-well plate) were mixed at a ratio of 1:1 and cultured in DMEM, containing 5% FBS, 100 U/ml penicillin and 100 mg/ml streptomycin.

The cells were grown until 70% confluent and were subsequently incubated with fresh DMEM. Following incubation for 24 h, the HCC-CM was collected and centrifuged at 600 × g for 10 min at room temperature to remove debris, filtered through a 0.2 mm filter (EMD Millipore, Billerica, MA, USA) and stored at −20°C until use.

### Proliferation assay

A 3-(4,5-dimethylthiazol-2-yl)-2,5-diphenyltetrazolium bromide (MTT) assay (Aladdin Industrial, City of Industry, CA, USA) was performed to detect the proliferation rate of the LX-2 cells. Briefly, 5×10^3^ LX-2 cells were seeded into a 96-well plate (Corning, Inc., Corning, NY, USA) for 24 h at 37°C and the cells were then starved for an additional 24 h in DMEM supplemented with 0.5% FBS. The medium was replaced with DMEM containing 2% FBS and different quantities of CM (10, 20, 30, 40, 50, 60 and 70%) or CD147 (0.125, 0.25, 0.5 and 1.0 *μ*g/ml) for 24 h. A total of 10 *μ*l MTT solution (10 mg/ml) was added to each well and incubated at 37°C for 4 hours. The media was then removed, 100 *μ*l dimethyl sulfoxide (Shanghai Ziyi Reagent Factory, Shanghai, China) was added to each well, and the plate was agitated for 10 min, in order to dissolve the crystals that had formed. The plate was then incubated at 37°C for 10 min, and absorbance was measured at a wavelength of 490 nm using an enzyme-linked immunosorbent assay detector (DG5031; Nanjing East China Electronics Group Co., Ltd., Nanjing, China). Cell proliferation was measured using an MTT assay based on the change in absorbance at 490 nm using the formula: (A_490_experimental − A_490_control) × 100% / A_490_control.

### Detection of metalloproteinase (MMP) secretion using gelatin zymography

Primary-cultured HSCs (5×10^3^) at passage two were seeded into a 96-well plate. At 70% confluence, the cells were starved overnight in serum-free DMEM at 37°C prior to the media being replaced with DMEM-supplemented 2% FBS and different concentrations of CD147 (0.125, 0.25, 0.5 and 1.0 *μ*g/ml). Following incubation for 24 h, the cells were cultured in serum-free DMEM for 24 h. The medium was harvested and the expression levels of the MMPs were measured using gelatin zymography, which was conducted as follows. Samples (40 *μ*l) to be tested were mixed with 2X Tris-glycine SDS sample buffer and were rested for 10 min at room temperature. The gel, which contained 0.1% Gelatin (Beijing YiRan Biological Technology Co., Ltd., Beijing, China), was ran with 1X Tris-glycine SDS running buffer. The gels were then incubated at room temperature with agitation in zymogram renaturing buffer for 30 min, then with zymogram developing buffer for 30 min. The buffer was then refreshed and it was incubated at 37°C overnight. The gels were then stained with 0.5% (w/v) Coomassie Blue R-250(Beijing YiRan Biological Technology Co., Ltd.) for 2 h, then were destained with Coomassie R-250 destaining solution until areas of protease activity appeared as clear bands. The Tris-glycine SDS sample/running buffers, the zymogram renaturing/developing buffers and Coomassie R-250 destaining solution were all prepared by the Environment Related Gene Key Laboratory of Ministry of Education (Xi’an, China). Densitometric analysis of the expression of MMP was performed using a calibrated GS-670 densitometer (Bio-Rad Laboratories, Inc., Hercules, CA, USA).

### Western blot analysis

The LX-2 cells were seeded into a 6-well plate and were treated with different concentrations of CD147 0.125, 0.25, 0.5 and 1.0 *μ*g/ml). Following 24 h treatment, the medium was removed and the cells were harvested using radioimmunoprecipitation (RIPA) cell lysis buffer (Beyotime Institute of Biotechnology, Nantong, China). For the tissue specimens, the liver tissues were collected from rat models of hepatic fibrosis induced by carbon tetrachloride (CCl_4_; The Third Chemical Reagent Factory, Tianjin, China). The tissues were cut into small sections and the cells were disrupted using a tissue homogenate method. Briefly, the tissue sections were added to RIPA, placed on ice, and subsequently homogenized using a pro200 Homogenizer (Pro Scientific, Inc., Oxford, CT, USA). The total protein was extracted from 50 mg tissue samples and LX-2 cells in a 6-well plate using RIPA cell lysis buffer, and the protein concentration was measured using a Bicinchoninic Acid Protein Assay kit (Beyotime Institute of Biotechnology). Western blotting was performed, according to a standard method.

### Reverse transcription-quantitative polymerase chain reaction (RT-qPCR) analysis

After treatment with 40% CM or 0.125, 0.25, 0.5 and 1 *μ*g/ml CD147, total RNA was extracted from the LX-2 cells using TRIzol reagent, according to the manufacturer’s instructions (Promega Corporation, Madison, WI, USA). cDNA was reverse transcribed from 1 μg total RNA, using ReverTra Ace-α™ kit (Toyobo Co., Ltd., Osaka, Japan). RT-qPCR analysis was performed using SYBR Green PCR Master mix (Applied Biosystems, Foster City, CA, USA), according to the manufacturer’s instructions, using a StepOnePlus™ Real-Time PCR system (Applied Biosystems). A total of 1.6 *μ*l template cDNA was used for amplification, and the PCR conditions were set at: Initial denaturation at 95°C for 30 sec, 95°C for 5 sec and 58°C for 30 sec for 35 cycles, then 95°C for 15 sec, 60°C for 1 min, 95°C for 15 sec and annealing and extension at 58°C for 30 sec. The gene expression levels of α-SMA, collagen I and TIMP were measured and compared against the expression of β-actin. The sequences of the oligonucleotides used are shown in Table I. The data were analyzed usign StepOne v2.3 software (Life Technologies, Carlsbad, CA, USA).

### Immunofluorescence staining

The FHCC-98 and LX-2 cells (1×10^5^ cells/6-well plate) were mixed and seeded into 6-well plates with coverslips (Huarui Medical Instrument Co., Ltd., Taizhou, China). Following co-culture for 1, 2 or 4 days, the cells were washed with 1X phosphate-buffered saline (PBS) and were fixed in cold acetone (Nanjing Chemical Reagent Co., Ltd., Nanjing, China) for 10 min at room temperature. The aspecific sites were blocked for 30 min using 2% bovine serum albumen (Shanghai Gaochuang Chemical Technology Co., Ltd., Shanghai, China) in PBS. The coverslips were incubated with mouse monoclonal primary antibodies against α-SMA (cat. no. ab7817) and CD147 (cat. no. ab78106), diluted 1:500 with blocking solution, overnight at 4°C. The control groups were only incubated with one of α-SMA or CD147. Following three washes in PBS, the cells were incubated with secondary antibodies conjugated to fluorophores (FITC, BA1101; Cy3, BA1031; Invitrogen Life Technologies) for 1 h at room temperature, following which, 4,6-diamidino-2-phenylindole (Sigma-Aldrich, St. Louis, MO, USA) was added to stain the nuclei. The coverslips were washed, as above, and then mounted (PBS-glycerin; Beijing Dingguo Changsheng Biotechnology Co., Ltd., Beijing, China) and observed using fluorescence microscopy (BX53 microscope and DP72 charge-coupled device camera; Olympus Corporation, Tokyo, Japan).

### Rat models of hepatic fibrosis

The present study was approved by the ethics committee of Xi’an Jiaotong University Laboratory Animal Center (Xi’an, China). Specific pathogen-free male Sprague-Dawley rats (n=10; weight, ~250 g) were supplied by the Experimental Animal Centre of the Fourth Military Medical University (Xi’an, China) and were maintained in a sterile room at 25°C with 40% humidity, a natural light/dark cycle and *ad libitum* access to food. The rats were randomly assigned into three groups: Control group, 4 weeks CCL_4_ treatment group and 8 weeks CCL_4_ treatment group. For the induction of liver fibrosis, the animals were intraperitoneally injected with 2 ml CCl_4_/peanut oil (Shandong Luhua Group Co., Ltd., Laiyang, China) solution (20% v/v) twice a week for 4 or 8 weeks (CCl_4_ group). The rats were administered 2 ml physiological saline (PS; 0.9% NaCl in ddH_2_O), replacing the CCl_4_, in the PS group. The rats were subsequently sacrificed by cervical dislocation following 4 or 8 weeks of CCl_4_ induction and with intraperitoneal anesthetization with 0.6 ml sodium pentobarbital (2% w/v; Shanghai XiTang Biotechnology Co., Ltd., Shanghai, China).

### Detection of α-SMA and CD147 by immunohistochemical staining

The liver tissues were fixed in 10% formalin (Xi’an Fuli Chemical Plant, Xi’an, China) at room temperature and embedded in paraffin (solid; melting point, 56–58°C) at 60°C Sections were then cut (5 *μ*m) from the tissues. Following deparaffinization by an ascending gradient of alcohol (Xi’an Chemical Reagent Factory, Xi’an, China) from 70% to 100% and hydration by a descending gradient of alcohol from 100% to 70%, the tissue sections were immunohistochemically stained, according to the manufacturer’s instructions. Briefly, the tissue sections were boiled for 20 min in citrate buffer solution for antigen retrieval and incubated for 10 min with 30% H_2_O_2_ in an 80% methanol solution to inactivate the endogenous peroxidases. Following washing three times with PBS, the liver tissue sections were incubated overnight at 4°C with primary antibodies against α-SMA (cat. no. ab7817) and CD147 (cat. no. ab78106), which were diluted 1:100 in blocking buffer (PBS containing 5% bovine serum albumin and 0.1% Triton X-100; Sigma-Aldrich). Following washing, as above, the slides were incubated with horseradish peroxidase-conjugated goat anti-mouse secondary antibody (cat. no. BA1004; 1:200) for 1 h at room temperature. Diaminobenzidine (DAB Horseradish Peroxidase Chromogenic Reagent kit; Beyotime Institute of Biotechnology) was added to the slides to visualize specific antigen, and hematoxylin (Shanghai Yuanye Biotechnology Co., Ltd., Shanghai, China) was used to stain the nucleus. Finally, the tissues sections were dehydrated, hyalinized sequentially and were then mounted for visualization (BX53; Olympus Corporation).

### Detection of α-SMA and CD147 in liver tissues by western blotting

Tissue samples (50 mg) were lysed in non-ionic detergent-containing buffer (RIPA lysis buffer). Following centrifugation, the protein concentration was determined using a bicinchoninic acid assay (Beyotime Institute of Biotechnology). The total protein (50 *μ*g) from each sample was subjected to 10% SDS-PAGE and transferred onto a polyvinylidene difluoride membrane (Bio-Rad Laboratories, Inc.). The membrane was blocked using 5% non-fat milk for 1 h at room temperature and was then incubated with mouse monoclonal antibodies against α-SMA (cat. no. ab7817) and CD147 (cat. no. ab78106) at 4°C overnight. Following washing with Tris-buffered saline containing 0.1% Tween-20, the membrane was incubated with horseradish peroxidase-conjugated goat anti-mouse (cat. no. BA1004; 1:500) antibody for 1 h at room temperature. Immunoreactive bands were visualized using an enzyme-linked chemiluminescence detection kit (GE Healthcare, Piscataway, NJ, USA).

### Statistical analysis

The data are expressed as the mean ± standard deviation of three independent samples. The results were analyzed using Student’s t-test for independent samples. P<0.05 was considered to indicate a statistically significant difference.

## Results

### Activation of HSCs is induced by HCC-CM

**Figure 1:**
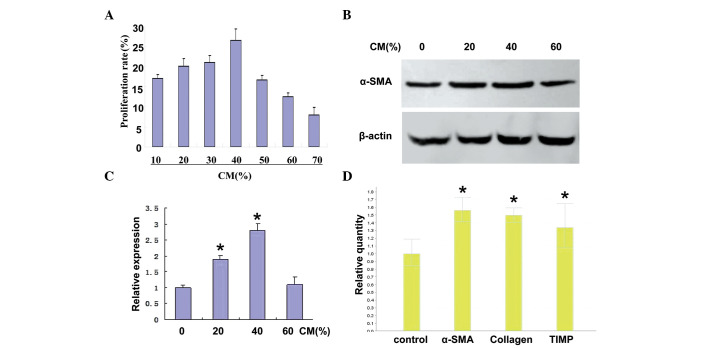
LX-2 cell activation is promoted by CM. (A) LX-2 cells were incubated with different concentrations of CM from FHCC-98 cells for 24 h and cell proliferation was measured using an 3-(4,5-dimethylthiazol-2-yl)-2,5-diphenyltetrazolium bromide assay. The proliferation rate was calculated as follows: (A_490_ experimental − A_490_ control) × 100% / A_490_ control. (B) Western blotting was used to detect the expression of α-SMA in human LX-2 HSCs following treatment with HCC-CM. β-actin was used as the internal standard for normalization. (C) Relative expression of α-SMA was measured using Quantity One density scanning software. (D) mRNA expression levels of α-SMA, collagen I and TIMP-1 were determined by RT-qPCR. The LX-2 cells were treated with 40% CM for 24 h and the cells were harvested. The total RNA was extracted and was subjected to RT and fluorescence qPCR. β-actin was used as an internal standard for normalization. The data are expressed as the mean ± standard deviation of three independent experiments (*P<0.05, compared with the control group). CM, conditioned medium; HSC, hepatic stellate cell; HCC, hepatocellular carcinoma; SMA, smooth muscle actin; TIMP, tissue inhibitor of matrix metalloproteinase; RT-qPCR, reverse transcription-quantitative polymerase chain reaction.

To determine the role of HCC-CM on the activation of the LX-2 cells, the viability of the LX-2 cells was assessed using an MTT assay following 24 h stimulation with CM. As shown in Fig. 1A, treatment with HCC-CM at concentrations between 10 and 70%, stimulated cell growth, however, cell viability was increased more markedly by lower concentrations of CM compared with higher concentrations. A significant increase in proliferation rate (25.8%) was observed following the addition of 40% CM.

The protein expression of α-SMA in human LX-2 HSCs following treatment with HCC-CM was investigated by western blotting. As shown in Fig. 1B, when the LX-2 cells were treated with 20, 40 or 60% HCC-CM, the expression of α-SMA increased compared with the untreated control cells. The highest expression level of α-SMA was detected in the LX-2 cells stimulated with 40% CM. The relative expression of α-SMA in the LX-2 cells treated with HCC-CM is shown in Fig. 1C.

RT-qPCR was performed to determine whether HCC-CM induced the activation of LX-2 cells. As shown in Fig. 1D, the expression levels of α-SMA, collagen and TIMP were upregulated in the LX-2 cells following treatment with 40% CM, suggesting that the LX-2 cells were activated following stimulation with HCC-CM.

### CD147 stimulates the activation of HSCs

**Figure 2:**
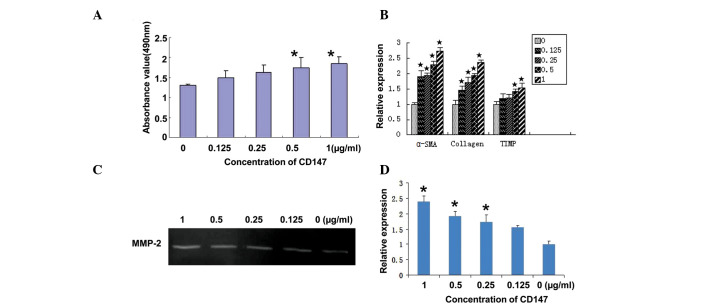
CD147 stimulates the activation of HSCs (A) LX-2 cells were incubated with different concentrations of CD147 (between 0.125 and 1 *μ*g/ml) for 24 h and the optical absorption value was measured using a 3-(4,5-dimethylthiazol-2-yl)-2,5-diphenyltetrazolium bromide assay. (B) mRNA expression levels of α-SMA, collagen I and TIMP-1 were determined by reverse transcription-quantitative polymerase chain reaction following treatment with CD147 at different concentrations. β-actin was used as an internal standard for normalization. (C) Secretion levels of MMP-2 in LX-2 cells were detected by gelatin zymography following stimulation of CD147 at different concentrations. (D) Relative secretion levels of MMP-2 were determined by scanning densitometry of the gelatin zymography. The data are expressed as the mean ± standard deviation of three independent experiments (*P<0.05, compared with the control group). CD, cluster of differentiation; MMP, matrix metalloproteinase; SMA, smooth muscle actin; TIMP, tissue inhibitor of MMPs.

The present study used CD147 to estimate its role in the activation of HSCs. LX-2 cell viability was detected using an MTT assay 24 h after stimulation with CD147. As shown in Fig. 2A, CD147 at concentrations of 0.5 and 1 *μ*g/ml promoted the proliferation of LX-2 cells in a dose-dependent manner, as expected, with a maximum proliferation rate of 32.5% following treatment with 1 *μ*g/ml CD147.

RT-qPCR was used to detect the expression levels of α-SMA, collagen I and TIMP in LX-2 cells following stimulation with CD147 at different concentrations. As shown in Fig. 2B, these genes were upregulated in a dose-dependent manner, suggesting that the LX-2 cells were activated following treatment with CD147.

Gelatin zymography was used to analyze the secretion of MMP-2 in primary HSCs following stimulation with CD147. The secretion of MMP-2 in LX-2 cells treated with CD147 at different concentrations increased compared with the control cells (Fig. 2C). The quantitative determination of MMP-2 by scanning densitometry of the gelatin zymography revealed a significant difference in the secretion of MMP-2 between the stimulated and non-stimulated cells (Fig. 2D; P<0.05).

### HCC cells induce the activation of HSCs

**Figure 3:**
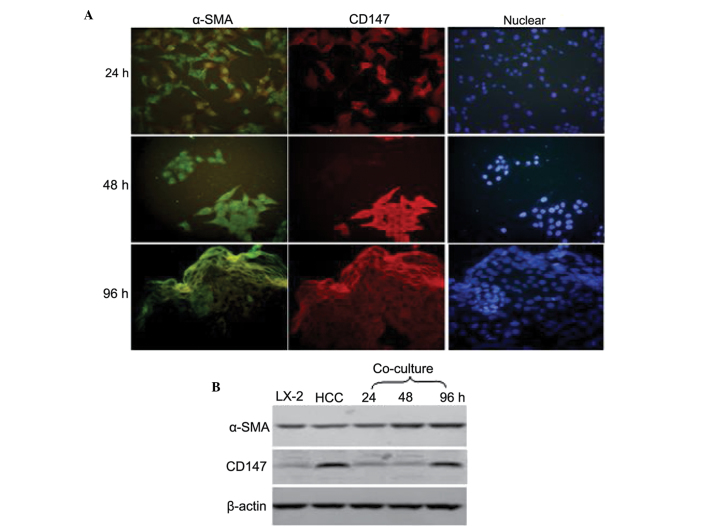
HCC cells induce the activation of HSCs (A) Immunofluorescence staining with 4,6-diamidino-2-phenylindole was used to detect the expression levels of α-SMA (green fluorescence) and CD147 (red fluorescence) in co-cultured HCC and LX-2 cells after 24, 48 and 96 h (magnification, ×200). (B) Expression levels of α-SMA and CD147 were investigated in the FHCC-98, LX-2 and co-cultured cells by western blotting. SMA, smooth muscle actin; CD, cluster of differentiation; HCC, hepatocellular carcinoma.

The HCC cells and LX-2 cells were co-cultured, as described above. The mixed cells were seeded into a 6-well plate with coverslips. Following culture for 24, 48 or 96 h, immunofluorescence staining was used to detect the expression levels of α-SMA and CD147, as described above. As shown in Fig. 3A, following co-culture for 24 and 48 h, the HCC cells expressed CD147 (red fluorescence) and the LX-2 cells expressed α-SMA (green fluorescence). Following co-culture for 96 h, the LX-2 cells expressed α-SMA and CD147, suggesting that the LX-2 cells were activated and the shape changed to that of fibroblasts.

To further investigate whether the expression of CD147 in the LX-2 cells was stimulated by HCC cells, the expression levels of α-SMA and CD147 were detected in HCC cells, LX-2 and co-cultured cells by western blotting (Fig. 3B). The LX-2 cells expressed α-SMA and CD147 on activation following co-culture with the HCC cells.

### CD147 is expressed in rat models of hepatic fibrosis induced by CCl_4_

**Figure 4:**
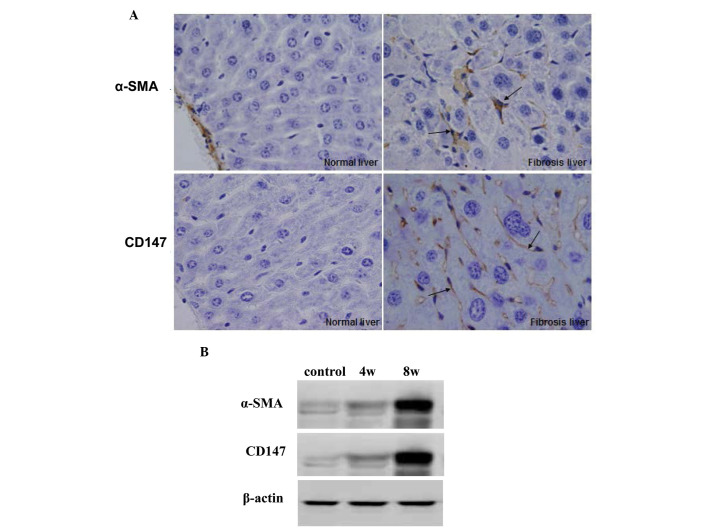
CD147 is expressed in rat models of hepatic fibrosis induced by CCl_4_. (A) Following treatment with CCl_4_ for 4 or 8 weeks, the model rats were sacrificed and the liver tissue was cut into tissue sections to detect the expression levels of α-SMA and CD147 by immunohistochemistry (magnification, ×400). Arrows indicate α-SMA and CD147 expression. (B) Western blotting was used to determine the expression levels of α-SMA and CD147 in the fibrotic rat liver tissue following treatment with CCl_4_ for 4 and 8 weeks. SMA, smooth muscle actin; CD, cluster of differentiation.

The present study investigated the expression of CD147 in the liver of rat models of hepatic fibrosis, induced by CCl_4_, to determine whether activated HSCs secreted CD147 Following treatment with CCl_4_ for 4 or 8 weeks, the model rats were sacrificed and the liver tissues were used to detect the expression levels of α-SMA and CD147 by immunohistochemistry and western blotting. As shown in Fig. 4A, following treatment with CCl_4_ for 8 weeks, significant increases in the expression levels of α-SMA and CD147 were detected in the CCl_4_-treated rat liver compared with the normal control rats. Western blotting demonstrated identical results in the rat hepatic tissues (Fig. 4B).

## Discussion

Liver disease is characterized by excessive deposition of ECM proteins (2,3). The excess deposition of ECM proteins disrupts the normal architecture of the liver, which alters the normal function and, ultimately, leads to pathophysiological damage (3,15). During the development of liver disease, HCC cells and HSCs secrete cytokines (16) and subsequently produce an inflammatory microenvironment and dynamic stroma in the ECM (10), in which these two cell types grow. Following liver injury, the HSCs undergo a complex transformation or activation process, in which the cells change from quiescent cells into myofibroblasts (6). This, in part, is characterized by the appearance of the α-SMA cytoskeletal protein, therefore, the expression of α-SMA has been considered as a useful marker of activated HSCs (17). Activated HSCs are also the primary cell type responsible for collagen synthesis during liver disease (5).

There are reciprocal interactions between HCC cells and HSCs during hepatocarcinogenesis. Numerous types of solid malignant tumor arise on a background of inflamed and/or fibrotic tissues, which are detected in >80% cases of HCC (18). The infiltration of α-SMA-positive HSCs in the HCC stroma suggests that activated HSCs are important in the occurrence and development of HCC in patients with cirrhosis (19). Previous studies have demonstrated that HSCs drive the progression of HCC (20,21). The role of HCC cells on the activation of HSCs has also been investigated (22,23), however, few studies have investigated the molecular mechanism underlying the reciprocal interactions between HCC cells and HSCs. The present study demonstrated that HCC increases the activation of HSCs, and that CD147 is a key molecule involved in the cross-talk between HCC cells and HSCs.

CD147, also termed EMMPRIN or basigin, is a transmembrane protein, which is important in the metastasis and progression of cancer via inducing the production of MMPs (24). Since this protein exhibits marked expression levels in several types of carcinoma, HAb18G/CD147 acts as a cancer-associated biomarker for the detection of cancer (25), and is an effective target molecule for its treatment (26). CD147 is also expressed highly in HCC and promotes metastasis and progression (27), however, the function of CD147 in the activation of HSCs remains to be elucidated.

In the present study, the LX-2 human HSC line, induced by tumor-CM from human HCC cells, exhibited phenotypic characteristics, including increased cell proliferation, secretion of MMP-2 and gene expression levels of α-SMA, collagen I and TIMP-1, which are also induced by CD147. The results suggested that the CD147 molecule, secreted by the tumor cells, was involved in the activation of HSCs. It has been reported that the recruitment and activation of rat HSCs are under the control of tumor cells (28), and that HCC cell stimulate the growth, migration and expression of pro-angiogenic genes in human HSCs (13), indicating a different manner of tumor-induced activation from the classic fibrosis type activation.

There was increased expression of CD147 in the membranes of HCC cells, and also in the LX-2 cells following co-culture with the HCC cells, with a change in shape of the LX-2 to that of fibroblasts, suggesting that HCC cells triggered the epithelial-mesenchymal transformation of the HSCs via the secretion of CD147, which led to further activation of LX-2 cells and the secretion of CD147. These results demonstrated that HCC cells stimulated the activation of HSCs and the expression of CD147 in the LX-2 cells. The increased expression of CD147 triggers the epithelial-mesenchymal transformation of HCC cells, leading to a more aggressive and invasive phenotype (9,10). The present study further detected the expression of CD147 in liver tissues from rat models of hepatic fibrosis induced by CCl_4_. Treatment with CCl_4_ for 8 weeks led to marked expression of CD147 in the liver tissue. Combined with the *in vitro* results, it was suggested that CD147 is a key molecule involved in the cross-talk between HCC cells and HSCs.

A previous study demonstrated that HCC cells stimulate the growth, migration and expression of pro-angiogenic genes in human HSCs (13). Another investigation revealed that the activation of cultured rat HSCs is induced by tumoral hepatocytes and fetal bovine serum (12). The present study demonstrated that HCC cells secreted CD147, promoted the activation of HSCs and induced the expression of associated genes. These results are consistent with a previous study, which suggested the same function of HCC cells during the activation and transformation of HSCs (14).

Previous studies have demonstrated that activated HSCs promote the development of HCC (9,10,20,21), and that HSC cross-talk in the liver results in a permissive inflammatory microenvironment, which drives the progression of HCC (7).

In conclusion, although sevceral proteins and growth factors contribute to HCC cell-HSC interaction, the present study demonstrated that CD147 contributed to this cross-talk and also affected the tissue microenvironment. This affected the biological properties of the HCC cells and HSCs, possibly inducing a different clinical outcome. These findings emphasize the requirement for novel therapies targeting different tissue microenvironment components.

## Figures and Tables

**Table I:** Primer sequences for reverse transcription-quantitative polymerase chain reaction.

Gene	Primer sequence (5′-3′)	Product size (bp)
α-SMA		
Sense	TTCGTTACTACTGCTGAGCGTGAGA	200
Antisense	AAGGATGGCTGGAACAGGGTC	
Collagen I		
Sense	AACATGACCAAAAACCAAAAGTG	253
Antisense	CATTGTTTCCTGTGTCTTCTGG	
TIMP-1		
Sense	AGACCTACACTGTTGGCTG	130
Antisense	GACTGGAAGCCCTTTTCAGAG	
β-actin		
Sense	TGCTGTCCCTGTATGCCTCTG	261
Antisense	TTGATGTCACGCACGATTTCC	

SMA, smooth muscle actin; TIMP, tissue inhibitors of metalloproteinase.

## Abbreviations

HCC: hepatocellular carcinoma
HSC: hepatic stellate cell
CM: conditioned medium
MTT: 3-(4,5-dimethylthiazol-2-yl)-2,5-diphenyltetrazolium bromide
α-SMA: α-smooth muscle actin
TIMP-1: tissue inhibitor of metalloproteinase-1
MMP-2: matrix metalloproteinase-2
ECM: extracellular matrix
FBS: fetal bovine serum
